# Influence of UTCP on the employment of female workers and the supply of labor force

**DOI:** 10.1371/journal.pone.0259843

**Published:** 2021-11-15

**Authors:** Mingming Li, Jiayun Wang

**Affiliations:** 1 Department of Economics and Business, Central European University, Vienna, Austria; 2 Department of Social Policy and Intervention, Oxford University, Oxford, United Kingdom; German University in Cairo, CZECH REPUBLIC

## Abstract

In recent years, China has introduced the Universal Two-Child Policy (UTCP), which means that a couple can have two children. The implementation of this policy may affect female employment. Based on this background, this work aims to the impact of UTCP on the number and employment of Chinese women workers, and find out the countermeasures for the adverse impact of the policy. Firstly, the role of the Propensity Score Matching-Differences in Differences model is introduced, and the average and dynamic effects of UTCP on women’s employment are discussed by using the Propensity Score Matching model. Secondly, the survey data on issues related to female employment after the implementation of UTCP from 2016 to 2020 is analyzed. Finally, a conclusion is drawn according to the survey data. The results demonstrate that the implementation of UTCP widens the income gap between men and women. Meanwhile, the younger the couple, the greater the income gap. Besides, the unemployment rate changes slightly after the introduction of the policy. As the growth rate of female income is significantly lower than that of men of the same age, UTCP has little impact on the employment of Chinese female workers, but has a great impact on the quality of employment. Among all the respondents, the proportion of employed men is higher than employed women, which is about 64% ~ 65%. However, it is still unknown whether age, education, family characteristics, nationality, occupations, and economic development of the province have a certain impact on female income, which is worth noting by follow-up research. On the whole, the full liberalization of the second child has little impact on the employment of female workers in China, but has a great impact on the quality of employment. The present work lays a foundation for the study of the impact of UTCT on female employment in future, and offers a certain reference for the further study of the impact of the policy on employment in the future.

## 1. Introduction

In recent years, the problem of population structure in China has become increasingly prominent. The demographic dividend brought by the baby boom in the last century has gradually disappeared, and the trend of population aging has become ever more obvious. This problem has been highly concerned by all sectors of society and government departments. In October 2015, the communique of the Fifth Plenary Session of the 18th Central Committee of the Communist Party of China proposed to fully implement the Universal Two-Child Policy (UTCP). This communique pointed out that to make the population structure develop healthily, adhere to the basic national policy of family planning, and continuously improve the population development strategy, a couple could have up to two children legally, to deal with the population aging problem in China.

Because women naturally have a unique position and role in household production activities, the human capital investment and labor market employment of women show phased characteristics. On the contrary, men usually implement continuous human capital investment activities, and they have high human capital stock. In addition to the difference in human capital caused by lifestyle differences, companies prefer human capital investment behavior of men. Therefore, in the labor market, companies generally respond differently to the human capital difference due to gender and prefer to hire male workers. The issue of female employment has always been a hot topic in relevant fields. After the publication of UTCP, employers are more cautious about the recruitment of women of childbearing age. Brauner-Otto et al. (2019) pointed out that the increase in female labor force participation in the paid labor market was one of the most obvious family transformation, and it had gradually become a global phenomenon [1]. Besides, although this might improve income and bargaining power of women, it would inevitably bring life pressure and reduce time with children. Therefore, the authors explored the impact of this transformation on children’s health by combining newly collected data on children’s health outcomes, quarterly data on female employment, and family and community data. After adjusting for a robust set of personal, family and community factors, they found that women tended to engage in more common but lower quality and lower income jobs due to wage labor. They concluded that due to the influence of family and children, women did have many vulnerable factors in employment. The research of Asongu and Odhiambo (2020) proves this on the other hand [2]. The authors assessed the impact of the development and popularization of information technology on female employment discrimination and female economic participation in a group of 42 South African countries from 2004 to 2014. They used Gini coefficient, Atkinson index and Parma ratio to rate female employment discrimination. Besides, they adopted Information Communication Technology (ICT) indicators, including mobile phone penetration, Internet penetration, and fixed broadband. Their survey results showed that ICT indicators needed to be raised above a specific threshold to improve gender economic participation and ameliorate inequality. Fu et al. (2020) emphasized that promoting women’s employment was not only the need for social and economic development, but also the historical mission of emancipating women [3]. The scholars utilized the sampling survey data of 1% population in Guangdong Province in 2015 and analyzed the survey results. They found that the non-agricultural employment rate of women in counties of Guangdong Province was low, and the non-agricultural employment rate of women in more than three quarters of counties was less than 50%. Moreover, from the perspective of industry, there were significant spatial differences among women. In the southeast, women were primarily engaged in the secondary industry, while in the central and western regions, women were mainly engaged in the tertiary industry. Besides, the family size, fertility, and the number of middle-aged and elderly family members had an excessively negative impact on the employment of women. To sum up, in the present highly competitive environment, due to the specific role of women in the family and society, they are inevitably limited by family and other factors in job search. The implementation of UTCP means that female workers need more time for parturition and lactation, which increases the employment cost of employers and creates a certain economic burden on enterprises. It has further expanded the disadvantages of women in job hunting. Although the state has issued some relevant protection policies for female workers, women will ineluctably encounter rejection for different reasons in the actual workplace. Specifically, women will have less opportunities to participate in enterprise training and skill training to a certain extent. In terms of job promotion, enterprises will prefer employees who are familiar with business processes, have certain strength, and can keep up with the pace of the enterprise. Secondly, due to the influence of the second child, the focus of female employees will gradually shift from the workplace to the family, and their time and energy used for work will gradually shift to the family. Therefore, their energy and ideas for work will decrease accordingly, resulting in low work efficiency and affecting their actual performance at work.

After the implementation of UTCP, women are facing increasingly serious employment problems, mainly manifested in the difference with men in wages and benefits, post discrimination, and the exclusion of women in recruitment. At present, academic research on the employment discrimination of infertile women, women with one child, and women with two children has gradually matured. Investigating the impact of the implementation of UTCP on women generally focuses on the fertility cost and fertility intention of women, or family factors and legal system, but there are few studies on employment discrimination against women caused by UTCP. Therefore, it is of great value to study whether UTCP aggravates or alleviates employment discrimination against women [4]. In recent years, the Differences In Differences (DID) model is mostly used for quantitative evaluation of public policy in econometrics. Based on the data obtained from the natural experiment, the DID model effectively controls the prior difference between research objects and effectively separates the real results of policy impact through modeling [5]. Propensity Score Matching (PSM) is a common research method of studying the influencing factors of a treatment, policy, or other events. If whether an event has been executed is simply used as a dummy variable to regress the population, the parameter estimation will be biased. Such a comparison is obviously unscientific. Therefore, the PSM method is adopted [6, 7].

In the subsequent contents, the real-time employment data of females after the implementation of UTCP are analyzed based on the Propensity Score Matching-Differences in Differences(PSM-DID) model. Then, in an empirical analysis, females constitute the experimental group, and the males with the same characteristics belong to the control group. After a comparison, it is concluded that UTCP has a great impact on the employment of female workers and the supply of labor resources in China.

## 2. Research model on the impact of UTCP on female employment

Female employment discrimination has always existed in the labor market, such as gender income difference, vertical occupational segregation, and horizontal occupational segregation. Individual differences such as human capital differences and work experience are the principal reasons for gender income inequality. In addition to individual differences, institutional factors are also vital reasons for female employment discrimination. Most studies on female employment discrimination are aimed at gender income differences, because various forms of discrimination can eventually be presented in gender income differences. Questionnaire survey method and PSM method are the mainstream research methods of discrimination at present. Existing related studies have certain reference significance for the present work to study female employment discrimination under UTCP. UTCP has experienced three stages of change, and the time interval of each stage is two years, which puts forward stricter requirements for the data in this work. Based on the above requirements, the data set of the China Family Panel Studies (CFPS) is selected as the survey data, covering 25 provinces and cities. The CFPS has conducted a detailed survey on society, economy, population, and education, which can provide sufficient data support for this research.

### 2.1 Female employment discrimination

#### (1) Data selection

The family planning policy in China has experienced three changes from the one-child policy instituted in the late 1970s. 1. Two-Child Policy (Couples both from a one-child family are allowed to have a second child). 2. Selective Two-Child Policy (Couples are eligible to have a second child when one spouse is an only child.) 3. UTCP (Every couple can have two children). The objective of the present work is to explore the employment discrimination against females at the stages of UTCP. The tracking survey data obtained by CFPS is selected for analysis, including the data of most provinces and municipalities across the country. The tracking data comes from surveys in 2014, 2016, 2018 and 2020, and the families have been tracked for a long time. Among the tracking survey data, addresses are the basis to ensure the smooth progress of the next visit, and they are also critical privacy messages. To protect the personal information of the interviewees, the data cannot publicize the names, addresses, company names, and other identifying information, and the addresses are re-encoded. After the data samples are preprocessed and the additional data is removed, there are 3,800 families in the tracking surveys from 2014 to 2020. The information that does not need secondary processing in the data contains names, genders, ages, educational backgrounds, and income, and information that needs to be processed again is registered permanent residence, the number of children, and the occupation. Besides, the province is used as a unit for data statistics, with the annual gross domestic product (GDP) of the provinces and cities added [8, 9]. Table 1 displays the method of processing the data.

**Table 1:** Initial data processing.

Data	Data Processing
Annual income	The data is converted to logarithmic form without the negative and zero.
Gender	Females form the experimental group and males froms the control group.
Age	Statistics only for people over 18 years old
Education	According to different academic qualifications, respondents are assigned to different positive integers. The uneducated is expressed as 1, people with a diploma in primary school are denoted as 2; those with a junior high school diploma are marked as 3; those with a high school diploma, a technical secondary school diploma, or a technical school diploma are denoted as 4; those graduated from universities, junior colleges, and higher vocational colleges and institutions are denoted as 5; masters are marked as 6; doctors are denoted as 7.
Nationality	Minorities are represented by 1, and Han nationalities are represented by 0.
Registered permanent residence	Rural is presented as 1, and urban is presented as 0.
Whether there is a second child	No is 0, and Yes is 1.
Occupation	Executives and senior technical personnel are expressed by 1, professional and technical personnel by 2, workers by 3, service industry practitioners by 4, and peasants by 5.
GDP of the provinces and cities	It is transformed into logarithmic forms.

#### (2) Analysis of employment discrimination

Occupational segregation refers to the significant difference in the occupational distribution between a group of workers and another group of workers. In other words, occupational segregation is the tendency of a certain profession or some professions to be dominated by individuals of a certain race or gender. Usually, the coefficient of variation is used to represent the degree of segregation. For example, ethnic minorities in America only get low-wage jobs when they are allowed to work, and better jobs are left to the members of the white privileged class [10–12]. If all occupations are isolated, the index is 100%. If all occupations are equally distributed, the index is 0%.

The main task of the study is to analyze the influence of UTCP on occupational segregation and income gap between males and females, with the influence factors of age, education, registered permanent residence, and whether there is a second child included.

### 2.2 DID

The DID model is a widely used measurement method to analyze the effect of policies in academic circles, which is used to evaluate the implementation effect of policies. The principal of this method is to regard institutional change and new policy as a “natural experiment” exogenous to the economic system, and then divide the samples into the control group and the treatment group. The results of the control group are used to represent the results that the treatment group is not affected by the policy. In other words, the result of the control group is used as the adverse results of the treatment group. Then, the estimation of policy effect is obtained through the difference between the results of the control group and treatment group. The DID model effectively solves the endogenous problems caused by selection bias and missing variables. Therefore, in this work, women are regarded as the experimental group, and men as the control group, and the growth of female income is regarded as the counterfactual result of the increase in male income. Correpsondingly, the change of policies and the emergence of new policies are taken as an external “natural experiment” of the economic system. The new policy is implemented for the experimental group, and not for the control group. According to the comparison of relevant information on the two groups, the change amount and change rate of each group after the implementation of the policy can be calculated [13, 14], to obtain the difference between the two groups of changes.

In summary, the DID model primarily has the following three advantages. Firstly, it can tackle the problem of endogeneity, and the fixed-effect estimation can deal with the problem of missing variables. Secondly, the traditional methods of mearsuring the policy generally set a dummy variable, that whether the policy puts in place, for regression analysis. By contrast, the DID model can evaluate the policy effect more scientifically and accurately. Thirdly, the DID model is simple and easy to understand and apply.

In this experiment, females are taken as the experimental group, and males are regarded as the control group. Meanwhile, the income growth of males is taken as the counterfactual results of income growth of females [15] to obtain Average Treatment Effect On The Treated (ATT) of UTCP, as shown in Eq (1).


$$ATT=[E(Y_{1i}^{T}|D=1)-E(Y_{1i}^{T}|D=0)]-[E(Y_{0i}^{T}|D=1)-E(Y_{0i}^{C}|D=0)]$$ (1)

In Eq (1), *ATT* shows the adverse effects of UTCP on female employment, and *E* represents the income expectations of the respondents. Besides, *D* indicates the dummy variable of UTCP, *D* = 1 indicates females in the experimental group, and *D* = 0 represents males in the control group. Moreover, *T* means the experimental group, *C* is the control group, and *Y*_li_ and *Y*_0i_ are the income after and before the implementation of the policy. $\left[E(Y_{1i}^{T}|D=1)-E(Y_{1i}^{T}|D=0)\right]$ denotes the difference between the experimental group and the control group after UTCP, and $\left[E(Y_{0i}^{T}|D=1)-E(Y_{0i}^{C}|D=0)\right]$ refers to the difference between the two groups before the policy. Then, the experimental data obtained from the two groups are compared to obtain the adverse effects of UTCP on female employment [16, 17].

Specifically, the DID model is defined as Eq (3).


$$Y_{it}=\alpha_{0}+\alpha_{1}du+\alpha_{2}dt+\alpha_{3}du\cdot dt+\varepsilon_{it}$$ (2)

In Eq (3), *du* is grouped dummy variables. If the individual *i* is influenced by the policy, the individual *i* belongs to the experimental group, and the corresponding value *du* is 1. If the individual *i* is not affected by the policy, then the individual *i* belongs to the control group, and the corresponding value *du* is 0. Besides, *dt* is a dummy variable of policy implementation, the value *dt* before policy implementation is 0, and the value *dt* after policy implementation is 1. *du*⋅*dt* is the interaction term between grouped dummy variables and policy implementation dummy variables, and its coefficient *β*_3_ reflects the net effect of policy implementation. Table 2 reveals the net effect before and after the implementation of the policy.

**Table 2:** Comparison of net effects before and after the implementation of the policy.

	Before the policy	After the policy	Difference
The experimental group	*β*_0_+*β*_1_	*β*_0_+*β*_1_+*β*_2_+*β*_3_	*β*_2_+*β*_3_
The control group	*β*_0_	*β*_0+_ *β*_2_	*β*_2_
Difference	*β*_1_	*β*_1_+*β*_3_	*β*_3_

Before the DID model is used for processing, it is necessary to ensure that all data meets the following conditions. First, the data should be divided into groups randomly, so that each respondent has equal opportunity to be selected in the process of data selection. Second, all samples in the control group are certain to be affected by UTCP in the experiment. Moreover, the dependent variables of the experimental group and the control group can be different, only with the same development trend. Third, the parallel trend assumption must be considered, because male income as the control group without any data processing will lead to selection bias. It is necessary to select the control group with similar characteristics and consistent development trends with the experimental group before DID analysis.

The robustness test of DID ensures that all effects are indeed caused by the implementation of the policy [18–20]. Besides, the effect is mainly manifested in the following two aspects.

- Test of common trends. This requires several years of data to draw a complete and correct conclusion.
- Even if the trends in the experimental group and the control group are the same before the policy, the concern should be given to whether there are other policies that affect the trends, i.e., changes in the trends of the experimental group and the control group after the implementation of the policy. This problem can be viewed as the exclusiveness of the action mechanism of the treatment variables on the output variables. In this regard, the placebo test is carried out to regress the experimental group. Specifically, the data in the year before the implementation of the policy can be selected for processing, and the data of the group that is not affected by the policy is taken as the experimental group to conduct regression analysis. If the regression results of DID estimators under different fictional modes are significant, the original estimation results may have errors. The regression analysis are conducted on two groups to confirm whether the research conclusions are consistent. The factor that is immune to the policy can be the explained variable for regression. If the results of the DID are still significant, the original estimation may have errors.

### 2.3 PSM-DID

The following advantages of the DID method are the main reasons for its wide application. In the DID method, the measurement model is simple and easy to operate, and the regression estimation method is mature. From the perspective of measurement, the DID method actually adds two virtual variables and their multipliers into the regression equation, which simplifies the method and improves the effectiveness. However, although there are increasing studies on the DID method in China, scholars seem to equate the DID method with the simple multiplication of two dummy variables. They neglect that the natural experiment is the fundamental of the DID method, and ignore the elementary premise of using the measurement model. Consequently, some dummy variables that are completely inconsistent with the basic conditions of natural experiments are regarded as natural experiments, and some sample data that is not suitable for the DID method is selected for regression and estimation. In fact, the concept of the natural experiment commonly used in Chinese economic circles is often called the quasi-experiment, which has subtle but significant differences. The sample selection and grouping of quasi-experiments are carried out artificially by the experimenters, while natural experiments are completely random natural events. Quasi-experiments cannot completely randomly select and group experimental objects in the experimental environment, so its sample selection and grouping can only try to close to random. Therefore, the PSM method is adopted here for supplementation, which is widely used in medical research and econometrics, to solve the problem of selecting deviations (i.e. controlling confounding factors). The propensity score distribution ratio uses the propensity score value to find one or more individuals with the same or similar characteristics from the control group, to minimize the interference of other confounding factors.

The principles of the PSM-DID method are as follows. First, females (the experimental group) with similar characteristics in the survey are selected to match with males (the control group). Then, the paired analysis is performed to control the differences in the influence on the experimental group and the control group before UTCP and reduce the bias caused by the sample selection and the impact of endogenous problems. From the previous data, it is found that UTCP has a significant impact on the population without a second child, but has no significant impact on the population having multiple children. Besides, UTCP has no negative effect on male employment. Therefore, male employment is selected as the control group in the study. Moreover, the DID method is used to investigate the net effect of UTCP on female employment. The specific steps are as follows. Firstly, the logit model is used to re-select the samples in the experimental group and the control group, and a male with the similar propensity score in the control group is selected for each female individual in the experimental group. Then, the paired analysis is conducted to remove the bias caused by the systematic error of the object selection after UTCP. Therefore, in addition to the differences in income levels between the experimental group and the control group, other factors, such as ages and regions, are the same. Then, the DID model is used to calculate and estimate the net effect of UTCP on female employment [21–23].

The rationale of DID is to obtain the DID statistics reflecting the effect of the policy by comparing the difference in the influence on the control group and the experimental group before and after UTCP. This rationale and the content of Table 1 are transformed into a simple model. At this time, it is only essential to consider the coefficient of the interaction item in the model, to obtain the desired net effect of UTCP [24–26].

The net effect of female employment is verified according to Eq (3).


$$Y_{it}=\beta_{0}+\beta_{1}\cdot DID+\beta_{2}\cdot t+\sum \beta_{X}\cdot Control+\varepsilon_{it}$$ (3)

In Eq (3), *Y*_*it*_ represents the net effect of female employment, *i* denotes the *i*-th respondent, and *t* refers to the annual monitoring. Besides, *DID* is a given value of *t*, *β* stands for the main parameter to be estimated, *Control* signifies a series of values converted into data in Table 1, and *ε*_*it*_ represents the intervene term.

The verification of the dynamic effect of UTCP ON female employment is shown in Eq (4).


$$Y_{it}=\beta_{0}+\beta_{1}\cdot t+\beta_{2}\cdot t+\sum \beta_{m}\cdot treated^{m}+\sum \beta_{X}\cdot Control+\varepsilon_{it}$$ (4)

In Eq (4), *t* refers to the time, and the value is set to 3. Meanwhile, *Control* denotes a series of values converted into data in Table 1, *ε*_*it*_ represents the intervene term, and *treated*^*m*^ signifies the year of *m* under tests. 2014 is taken as 1, 2016 is taken as 2, and 2018 and 2020 are taken as 3.

Eq (5) indicates the Logit model constructed here.


$$Logit(X)=\beta_{0}+\sum \beta_{i}X_{i}+\varepsilon_{i}$$ (5)

In Eq (5), *X* represents the females of the experimental group affected by employment discrimination after UTCP, and *X*_*i*_ denotes the covariate among the females in the experimental group.

PSM is a statistical method used to process the data of observational studies. In the observational study, there are many bias and confounding variables due to various reasons. The PSM method can exactly reduce the influence of these bias and confounding variables to make a more reasonable comparison between the experimental group and the control group. The average processing effects obtained by different matching methods are extremely similar. Therefore, the nearest neighbor matching method is adopted here to deal with samples according to the ratio of 1:1, to obtain the experimental group and the control group [27, 28].

### 2.4 The hypothesis of t-test

The t-test, also known as student’s t-test, is mainly used for the normal distribution with unknown overall standard deviation σ of the sample. The t-test uses the t-distribution theory to infer the probability of difference and judge whether the difference between the two averages is significant. Eq (6) indicates the single overall test, and Eq (7) represents the double overall test.


$$t=\frac{\bar{X}-\mu }{\frac{\sigma_{X}}{n^{\frac{1}{2}}}}$$ (6)

$$t=\frac{\bar{X_{1}}-\bar{X_{2}}}{\sqrt{\frac{(n_{1}-1)S_{1}^{2}+(n_{2}-1)S_{2}^{2}}{n_{1}+n_{2}-2}(\frac{1}{n_{1}}+\frac{1}{n_{2}})}}$$ (7)

Among Eqs (7) and (8), *i* = 1,⋯,*n* and $\bar{X}$ are the average values of the representative samples, as shown in Eq (23). Besides, *S* denotes the standard deviation of the sample, *n* represents the number of samples, and *μ* refers to the average value of the overall distribution. Moreover, $S_{1}^{2}$ and $S_{2}^{2}$ are the variance of the two samples, and *n*_1_ and *n*_2_ are the capacity of two samples.

## 3. Impact of UTCP on female employment in China

### 3.1 Female labor force and the birth rate

#### (1) Female labor supply

**Fig 1:**
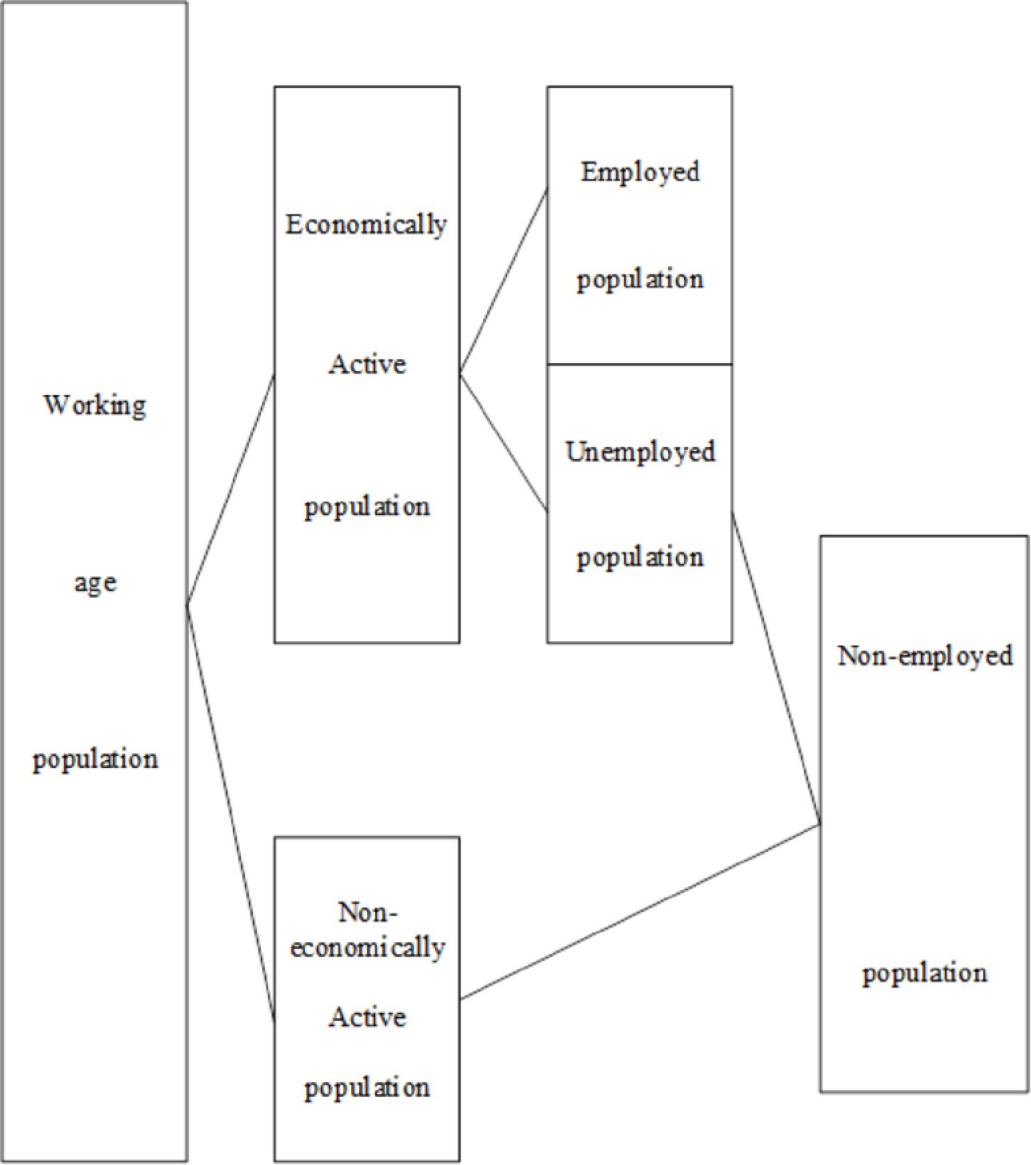
Classification of working-age population according to the international labor organization.

Labor supply is defined as the number of labor that decision-making subjects (individuals, families) of labor supply are willing and able to provide under the condition of a fixed market wage rate. Fig 1 shows the classification of working age populations by the International Labor Organization.

In 1978, the number of female workers in China was 170 million. In 1982, the number was 200 million, increasing by 30 million in four years. In 1990, the number was 290 million, with an increase of 90 million over the past eight years, which was the fastest-growing rate of female workers in China, and the rate gap of gender difference in the labor force was 12%. In 2000, there were 330 million female workers, increasing by 40 million in 10 years. In 2010, 2014, and 2016, the number stayed at 340 million. In 2018, the number was 350 million, and the female labor force participation rate exceeded 60%. The difference in the female labor force participation rate caused by gender differences reached 14.8%, which was slightly higher than that in 1990.

The proportion of women in various industries in 2020 is as follows: 15.5% are engaged in automobile / production / processing / manufacturing industry, 13.4% in IT / communications / electronics / Internet industry, 11.5% in trade / wholesale /retail / leasing / fast-moving industry, 9.6% in the service industry, 4.7% in the business service industry, 4.2% in the financial industry, 3.9% in stylistic education/arts and crafts industry, 3.5% in energy / mineral / environmental protection industry, 3.4% in transportation/ logistics industry, 1.1% in agriculture, forestry, animal husbandry, and fishery, and 6.8% in other industries.

#### (2) Newly-born population

**Fig 2:**
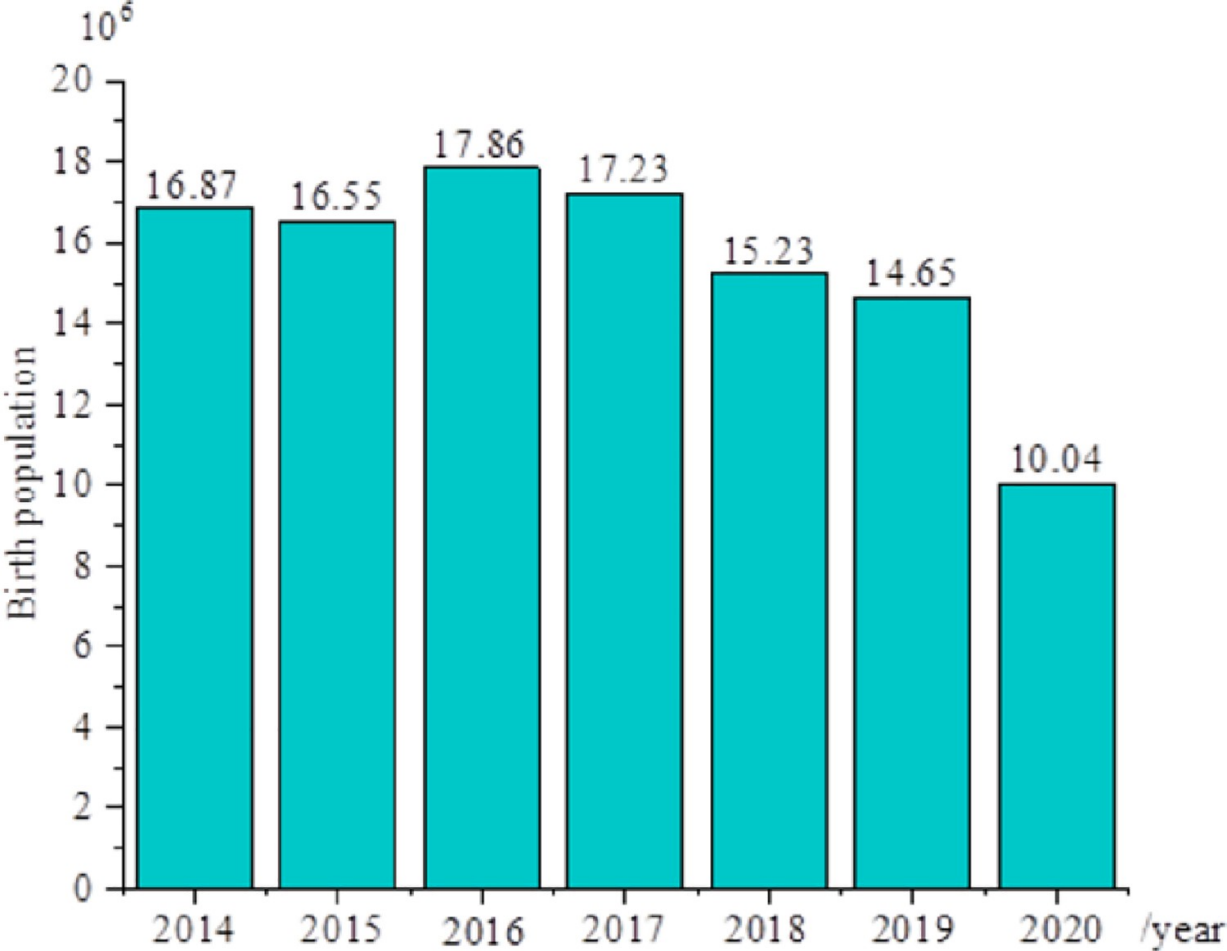
Birth population during 2014–2020.

**Fig 3:**
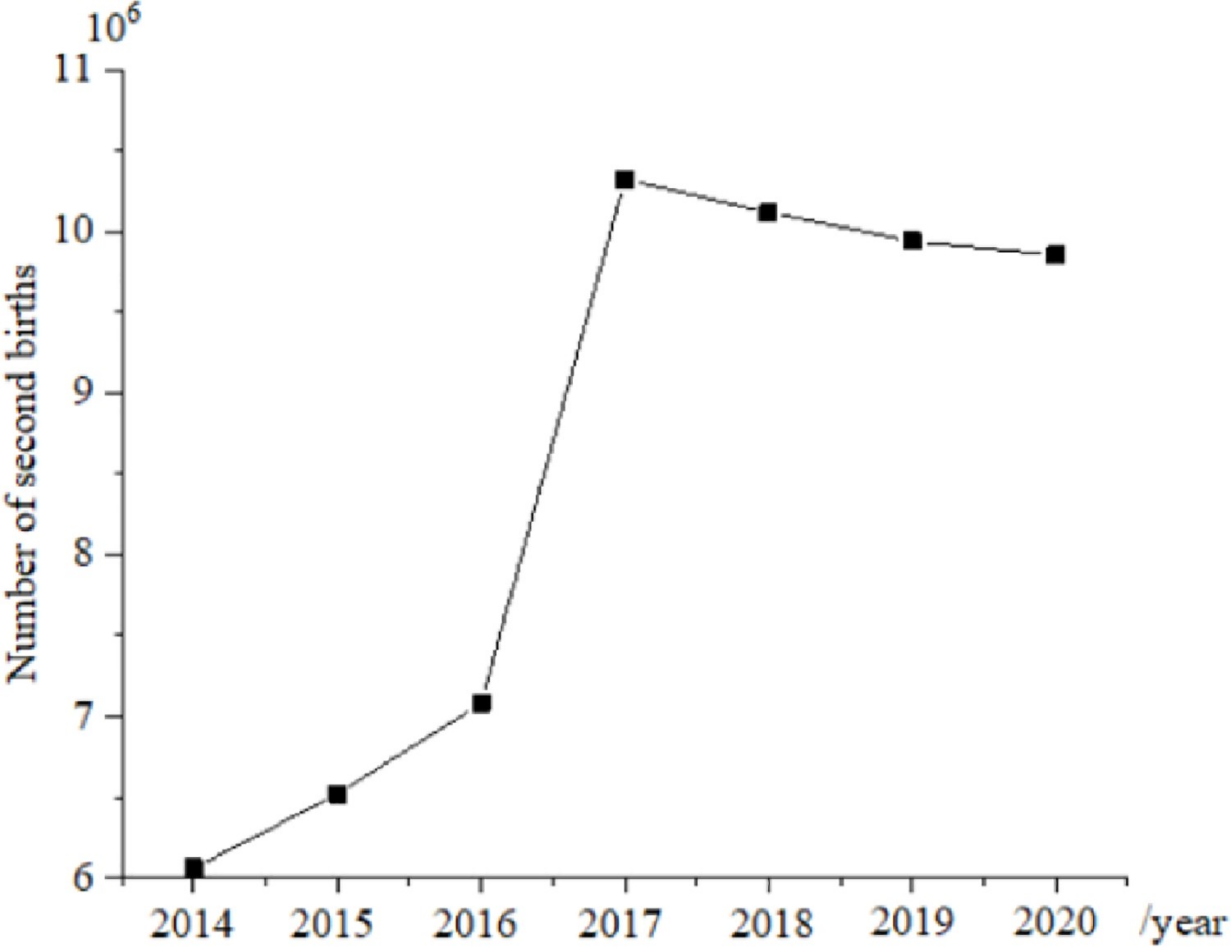
Number of second births during 2014–2020.

**Fig 4:**
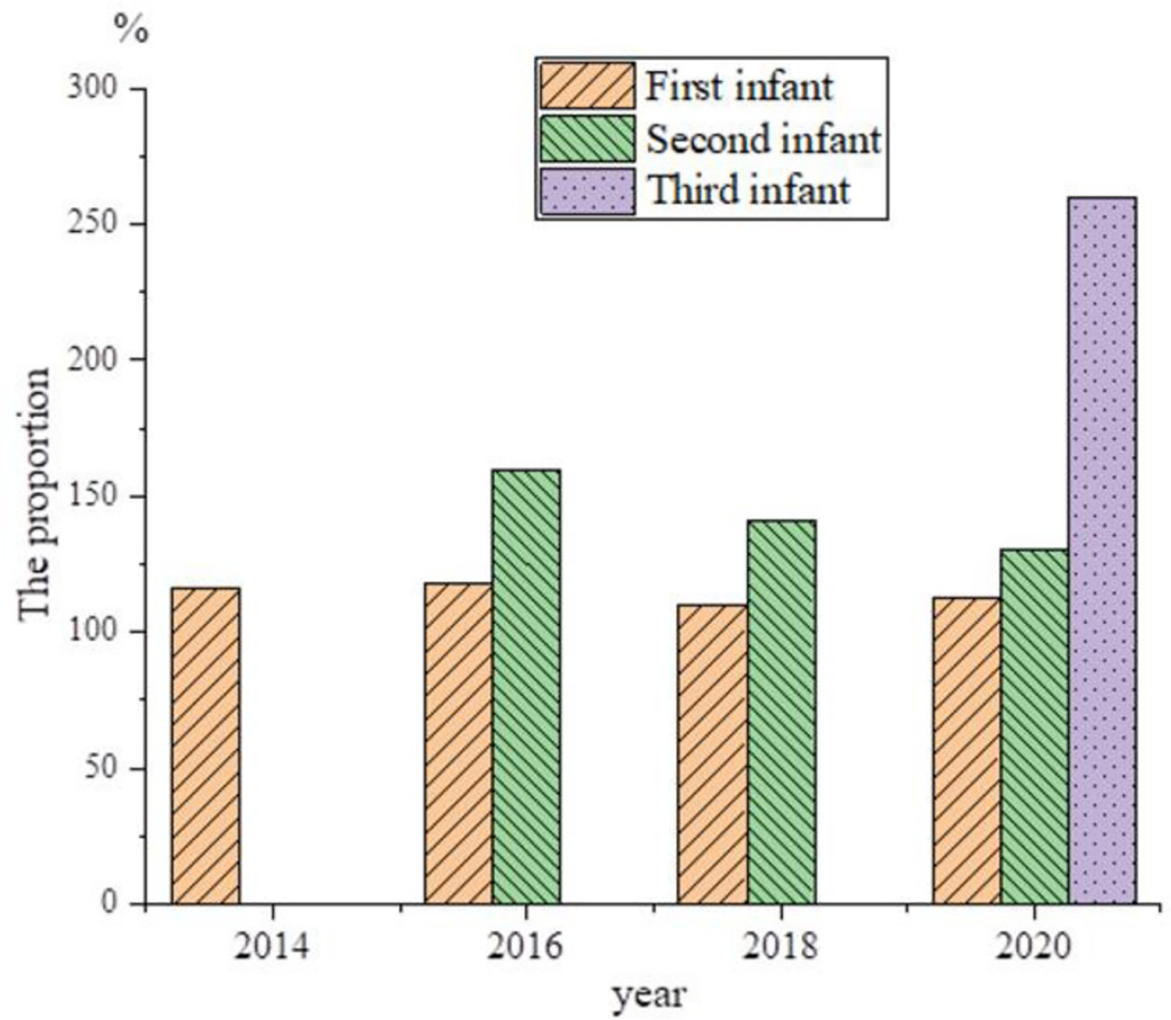
Sex ratio of birth population.

Fig 2 illustrates the newly-born population during 2014 ~ 2020, and Fig 3 displays the number of second births during 2014–2020. Fig 4 shows the sex ratio at birth in 2014, 2016, 2018, and 2020.

In 2017, the number of second births increases sharply by 10.325 million, and the growth rate is significant. However, the newly-born population begins to decrease year after year, and most of the newly-born population are second births. From 2017 to 2020, the birth population decreases five times, and there are only 10.4 million newborns in 2020. It is expected that the number of newborns will fall below 10 million in 2021, and the number of second births begins to decrease year by year.

### 3.2 Basic information of respondents

#### (1) Analysis of main data

Table 3 reveals the proportion of male respondents and female respondents, annual income, academic education, occupation, whether there is a second child, registered permanent residence, nationality, and the degree of development of the located provinces and cities from 2014 ~ 2020.

**Table 3:** Principal data of respondents.

Total number: 3,800	2014		2016		2018		2020	
	males	females	males	females	males	females	males	females
Proportion (%)	64.21%	35.79%	64.76%	35.24%	64.56%	35.46%	64.25%	35.75%
Annual income	21,962 CNY	17,341 CNY	31,905 CNY	23,868 CNY	37,412 CNY	26,380 CNY	39,495 CNY	27,557 CNY
Average age	42.72	39.32	44.65	41.34	46.78	43.41	48.81	45.41
Average GDP	3164.4 billion CNY		3567.1 billion CNY		3679.2 billion CNY		3891.3 billion CNY	
Proportion of receiving university education	16.13%	23.92%	17.16%	24.01%	18.27%	25.23%	19.34%	26.42%
Average proportion of executives	14.28%	15.02%	14.98%	16.12%	16.77%	21.74%	16.77%	21.75%
Average proportion of technicians	14.32%	20.46%	15.71%	18.62%	16.31%	21.22%	16.31%	18.62%
Average proportion of workers	39.51%	24.87%	55.92%	32.66%	39.64%	19.09%	39.62%	21.14%
Average proportion of practitioners in service industry	9.74%	29.64%	14.21%	32.46%	12.75%	28.36%	12.75%	29.38%
Average proportion of peasants	20.34%	14.67%	2.62%	3.17%	15.69%	11.43%	15.42%	11.41%
Average proportion of working in agricultural industry	49.34%	47.62%	48.17%	46.51%	47.98%	44.72%	47.53%	42.17%
Average rate of having two children	42.91%	32.01%	42.98%	32.16%	43.01%	32.18%	43.93%	43.03%
Average proportion of the minority	3.89%	4.01%	0.11%	0.31%	0%	0%	0%	0%

The proportion of male workers among all respondents is high, occupying 64% ~ 65%, while that of female workers is low, accounting for 35% ~ 36%. Since the PSM-ID model has high matching efficiency, it reflects the characteristics of female employment and increases the matching rate. Table 3 indicates that the income of males is significantly higher than that of females, and the income gap between males and females increases year by year. In addition, the average age of males is slightly older than that of females, with a gap of around 3 years old. From the annual GDP of the provinces where the men and women of the surveyed families belong, the average economic development level of the provinces where female workers are located is higher than that of the regions where male workers are located. Besides, the proportion of female workers with college education is higher than that of male workers, which shows that the fairness of education is relatively high. From the perspective of post distribution, there is no great difference in the proportion of men and women in senior managers, technicians, ordinary workers, farmers, and other posts, but nearly 50% of male workers are ordinary workers, while this proportion is only about 20% for women. Moreover, female employees account for about 30% of grass-roots practitioners in the service industry, while male employees account for less than 10%. Among all the respondents, the proportion of men among farmers is less than 50%, which is slightly higher than that of women. Furthermore, the proportion of male workers with a second child is also 10% higher than that of female workers. Among all respondents, the proportion of both men and women in ethnic minorities is very small, which is not worth discussing.

### 3.3 Empirical analysis of female employment discrimination

#### (1) Analysis of empirical results through the PSM model

**Fig 5:**
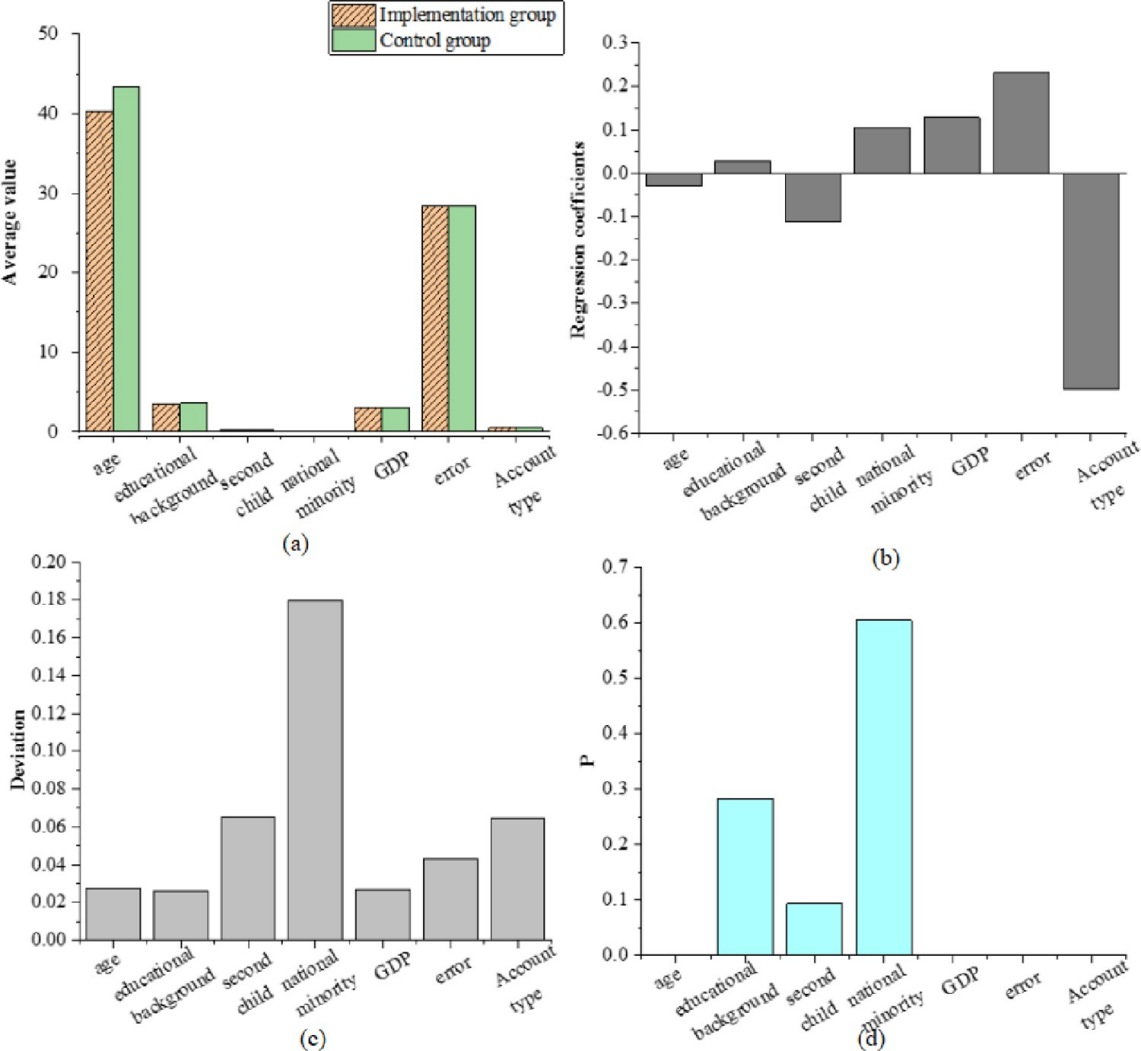
Matching results by nearest neighbor matching method (a) mean (b) regression coefficient (c) error (d) P.

Suitable samples in the periods of 2014 ~ 2016, 2016 ~ 2018, and 2018 ~ 2020 are selected as the control group to match the experimental group, to conduct the empirical analysis based on the Logit model. The data from 2014 to 2016 is used to obtain the regression results by the nearest neighbor matching method, as presented in Fig 5. In Fig 5, the X-axis variable parameters come from the age, education, with a second child, being ethnic minorities, average GDP, occupation, registered permanent residence in Table 3.

In terms of the matching results, the mean value of 2,405 samples of the experimental group is 9.531, and the mean value of 4,617 samples of the control group is 9.531. After the application of the nearest neighbor matching for propensity score, the error of all other data is less than 10% except whether they are ethnic minorities. The test results cannot refute the basic assumption that there is not a systematic difference in the influence on the experimental group and the control group. Therefore, the comparison with the results before matching is conducted, and the standardized deviation of most variables is significantly reduced by 70%, with the maximum reduction of 96.1%. The average annual income of the experimental group is 9.31, and that of the control group is 9.92. Moreover, the average effect is −0.4517, which is obvious at the level of 1%. P represents the change rate.

**Fig 6:**
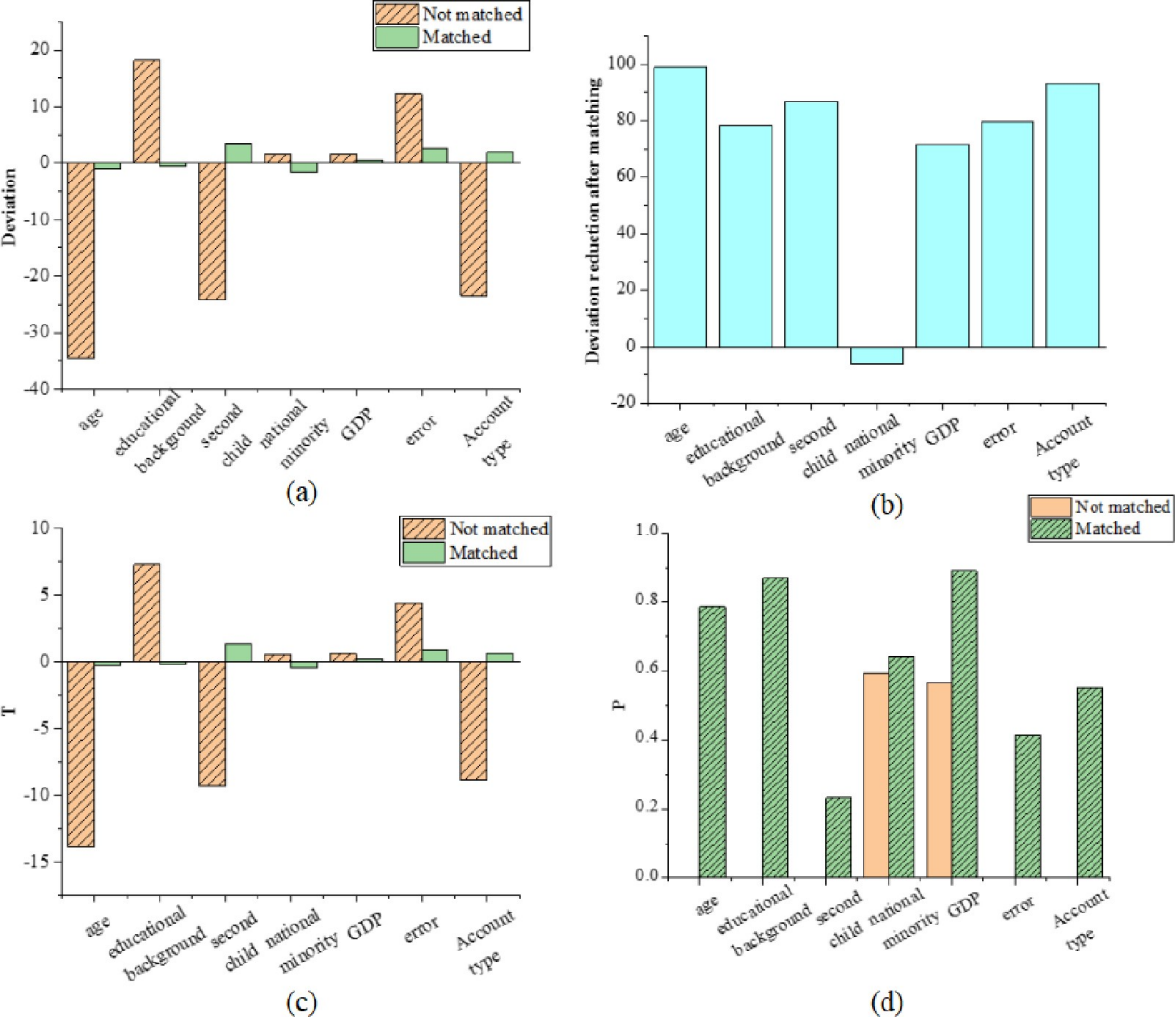
Results of a statistical test of propensity score matching quality (a) mean (b) regression coefficient (c) error (d) P.

Fig 6 displays the results of the statistical test of the propensity score matching quality.

In Fig 6, T is the statistic, and P is the change rate. Through the data matching and comparison between the experimental group and the control group, the error caused by systematic error is sharply reduced, the difference between them is an order of magnitude, and the age error decreases 98.8%. Besides, the impact of educational background decreases by 98.2%, the effect of whether there is a second child decreases by 86.7%, and impact of whether there are ethnic minorities decreases by 5.2%. As mentioned above, the impact of the last factor is extremely small, so the error rate changes little from positive to negative. Moreovver, the impact of occupation types decreases by 70.5%, the impact of annual GDP decreases by 79.5%, and the impact of the registered permanent residence decreases by 93.1%.

**Fig 7:**
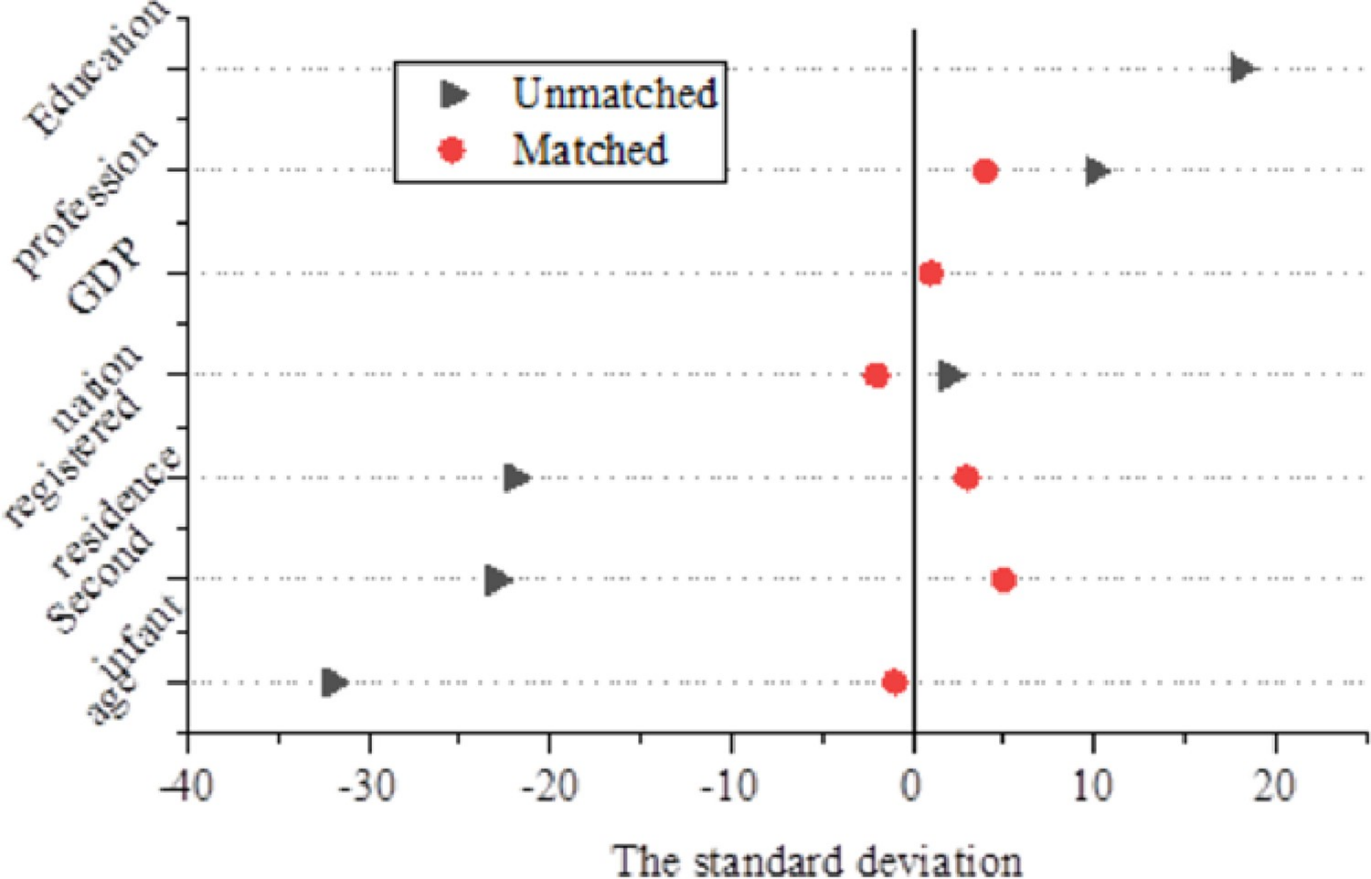
Changes in the standard deviation of the indicators matched.

**Fig 8:**
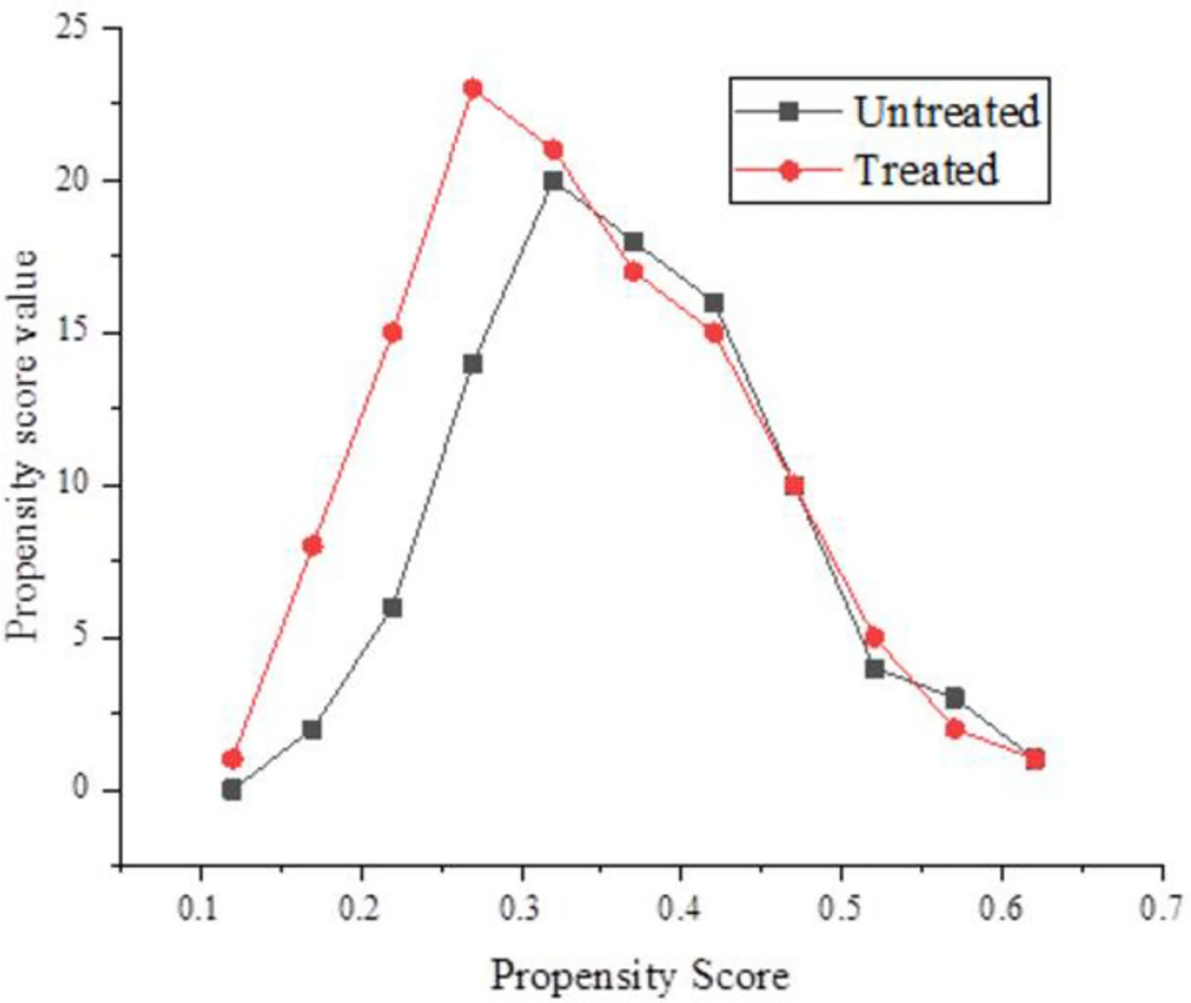
Range of propensity scores.

Fig 7 shows the variation of standard deviation of matching indicators, and Fig 8 provides the common range of the propensity score.

After the matching is conducted, except for ethnic variables, the standard deviations of all other variables are less than 10%, and the test results cannot reject the original hypothesis that there is no systematic difference between the experimental group and the control group. Compared with the results before matching, the standardization deviation of most variables is greatly reduced, the error reduction is more than 70%, and the maximum reduction is 97.7%. The average number of income logarithms in the experimental group is 9.419,543,2, and that in the control group is 9.862,818,42. The average treatment effect ATT is − 0.4433, which is significant at the level of 1%. The standard deviation of each variable after the matching is significantly lower than that before the matching. A common support test is also needed before the average processing effect is estimated. According to, the propensity score of the experimental group and the control group has a large enough overlap area within the range of values, namely the common support area. After the matching is conducted, the experimental group is in the common support area, and only 47 samples in the control group fall outside the common support area, so the matching results ensure the effectiveness of the propensity score matching.

#### (2) Empirical analysis of PSM-DID

An empirical analysis is performed on the influencing factors of the change of the income gap between different genders before and after the full liberalization of UTCP. Analysis results demonstrate that the full liberalization of UTCP will further improve the income gap between different genders. Besides, with time, the benefits of the policy will continue to increase, so the income gap between different genders will continue to increase. Whether for male or female, having multiple children has a huge negative impact on income, and farmers have obvious disadvantages. However, it is obvious that the impact on income caused by the nature of the industry and different occupations is becoming weaker and weaker, indicating that the income difference between different occupations is narrowing. UTCP has the strongest impact on the income level of the population of childbearing age, but it has little impact on the population of non childbearing age. In particular, female income level increases with the increase in education level, and policies to protect female employment are conducive to investment in education of women. Since bearing children will reduce the income of women of childbearing age, it is essential to strengthen the protection of women of childbearing age. Moreover, UTCP has a significant expanding effect on the income difference between men and women, and the difference is expanding with the passage of time and the strengthening of policy effectiveness. The impact of UTCP on the income difference between men and women is more significant for people of childbearing age, but not for people of non-childbearing age. The impact of UTCP is only aimed at employees of childbearing age. Hence, women of this group, that is, women of childbearing age, are worth employment protection. Specifically, the improvement of education can increase female income level, and female employment protection policies should encourage female education investment. Besides, bearing several children will reduce the income of women of childbearing age, so employment discrimination against them should receive severe punishment. Women of childbearing age in rural areas should also be paid attention to, since their income decline will enlarge. In terms of education level, UTCP has a more significant impact on the income difference between men and women without higher education, but has no impact on people with higher education. People with higher education generally have high productivity. Even if the implementation of UTCP potentially increases the cost of employing women, the high labor productivity of these people makes enterprises willing to hire women and give fair remuneration. In addition, the impact of age on higher education population is similar to that of other populations, with only a small impact, indicating that with the increase of age, the income of people with any education background will not change greatly. Therefore, people without higher education while with multiple children will face significantly decrease in income, so their employment protection should be strengthened. UTCP only slightly reduces the income of people with higher education, even those who are engaged in more ordinary occupations. Therefore, ordinary occupations should pay more attention to people without higher education. Women without higher education in rural areas should also be paid attention to, since their income is always low.

## 4. Conclusion

The impact of UTCP in China on female employment and the supply of female labor force in the market is studied in this work. Firstly, the number of births and the number of second births from 2014 to 2016 are analyzed, and the changes of female labor resource supply during this stage in China are studied. Then, a follow-up survey is conducted on 3,800 people in the seven years from 2014 to 2020 to collect various data on the impact of second births on these people. Then, these influencing factors are processed by the PSM-DID model to analyze the reasons and extent of the impact of UTCP on female employment and female labor supply. The experimental results indicate that UTCP will further enlarge the income gap between men and women, and the gap increases with time. On the contrary, the impact on income caused by different industries and occupations is becoming smaller and smaller. The policy has the greatest impact on the income of people of childbearing age. This work ultimately achieves research purpose of studying the impact of UTCP on female employment in China and the supply of female labor force in the market, and measures the net effect of UTCP on female employment discrimination through an empirical model. Besides, reasonable policy measures are put forward according to the research conclusions, which lays a foundation for relevant research on the liberalization of family planning in the future. Although the original expected research objectives and some valuable research conclusions are realized, due to limited academic literacy, there are still some deficiencies in the research work. The research conclusions may be limited by the following two factors. (1) The PSM-DID model is not perfect. (2) The data sample is not large enough, and there may be deviation since China has a vast territory. On the one hand, the follow-up study will employ a variety of models for data analysis. On the other hand, it is worth collecting more tracking survey data from different regions and provinces for comprehensive research and analysis.

## Supporting information

S1 Dataset (ZIP)
